# P-1988. COVID-19 vaccine induced antibodies in human milk: exploring form and function

**DOI:** 10.1093/ofid/ofae631.2146

**Published:** 2025-01-29

**Authors:** Amro Nasser, Dylan J Tuttle, Maris Wilkins, Priscilla Da Silva Castanha, Ernesto T A Marques, Anne-Marie Rick

**Affiliations:** University of Pittsburgh, Pittsburgh, Pennsylvania; University of Pittsburgh, Pittsburgh, Pennsylvania; University of Pittsburgh, Pittsburgh, Pennsylvania; University of Pittsburgh, Pittsburgh, Pennsylvania; University of Pittsburgh/ Fiocruz, Pittsburgh, Pennsylvania; University of Pittsburgh, Pittsburgh, Pennsylvania

## Abstract

**Background:**

Following SARS-CoV-2 vaccination/infection of pregnant/lactating women, human milk contains SARS-CoV-2 antibodies (Ab). Limited data exists on whether those Ab induce protection for infants against COVID-19. Among 45 lactating women who received primary and booster SARS-CoV-2 vaccines from 2021 to 2022, we previously showed that anti-receptor binding domain (RBD) IgG and IgA significantly increased in serum and milk following booster SARS-CoV-2 vaccine but did not differ by maternal nucleoprotein (NP) status. In this exploratory analysis, we aimed to further characterize the Ab present in human milk following maternal booster SARS-CoV-2 vaccination.

SARS-CoV-2 Neutralization Antibodies in Human Milk by Maternal Nucleoprotein Status before and after SARS-CoV-2 Booster Vaccine.

**Figure:**
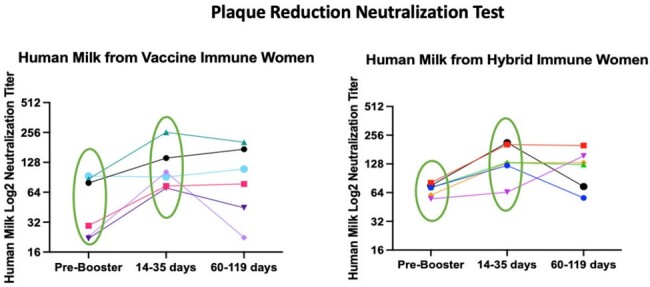

Neutralization titers in human milk significantly increased among women with hybrid immunity (NP+) after booster vaccine (P=0.04) but not for women with vaccine only immunity (NP-).

**Methods:**

Using a subset of paired longitudinal milk and blood samples from a cohort of 45 lactating women who received primary and booster SARS-CoV-2 vaccines from 2021 to 2022, we characterized neutralizing Ab, RBD IgG subclasses (IgG1-IgG4), and Ab-dependent complement activation (ADCA).

Antibody-Dependent Complement Activation (ADCA) using Human Milk from Lactating Women before and after SARS-CoV-2 Booster Vaccine.

**Figure:**
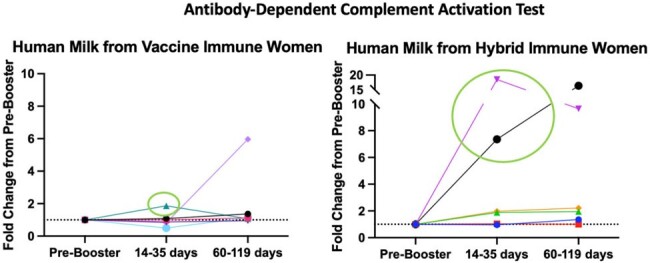

Two women with hybrid immunity (NP+) had significantly positive ADCA after SARS-CoV-2 booster vaccine, human milk from all others resulted in low or no activation of complement.

**Results:**

For 7 NP+ (hybrid immune) and 7 NP- (vaccine immune) women, SARS-CoV-2 neutralization titers increased in serum and milk after booster vaccine among NP+ women (p=0.04) but not for NP- women (Fig1). After booster vaccine, ADCA was positive but variable in maternal serum and milk with only 2/12 (16%) women demonstrating high-levels of ADCA in human milk (Fig2). These same two women demonstrated different IgG subclass profiles in milk after booster vaccine with moderate to high levels of IgG1 and IgG3, and low levels of IgG4 (Fig3). This was inverse for all other women who demonstrated moderate to low levels of IgG1 and high titers of IgG4 in human milk. Notably, both women with high ADCA response were NP+ and vaccinated with primary SARS-CoV-2 vaccine more than 4 months post-partum, while all others received primary SARS-CoV-2 vaccine while pregnant or less than a month post-partum.

IgG Subclasses in Human Milk before and after SARS-CoV-2 booster vaccine by Complement Activation Status

**Figure:**
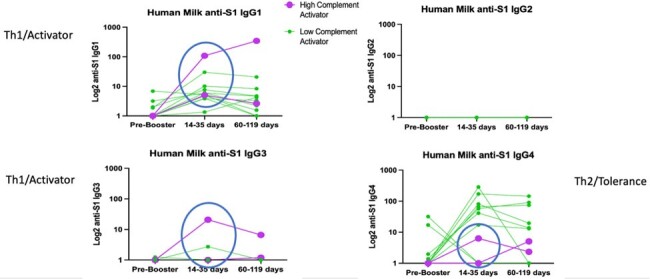

Human milk with high complement activation demonstrated higher levels of IgG1 and IgG3 after booster vaccine compared to women with low complement activation.

**Conclusion:**

The different Ab profile may indicate a tolerizing response that could be related to pregnancy at time of initial antigen exposure or from repeat SARS-CoV-2 vaccination and warrants future investigation (additional samples are under analysis). Nonetheless, human milk demonstrates robust binding and neutralizing Ab following SARS-CoV-2 vaccination, offering other potential mechanisms for protection of infants against COVID-19 infection.

**Disclosures:**

Anne-Marie Rick, MD, MPH, PhD, Pfizer: Advisor/Consultant

